# Remote underwater video reveals higher fish diversity and abundance in seagrass meadows, and habitat differences in trophic interactions

**DOI:** 10.1038/s41598-019-43037-5

**Published:** 2019-04-29

**Authors:** Salvador Zarco-Perello, Susana Enríquez

**Affiliations:** 1 Unidad Académica de Sistemas Arrecifales Puerto Morelos, Instituto de Ciencias del Mar y Limnología, Universidad Nacional Autónoma de México, Apdo. Postal 13, Cancun, 77580 Mexico; 2 School of Biological Sciences and UWA Oceans Institute, The University of Western Australia, Crawley (Perth), 6009 Western Australia, Australia

**Keywords:** Biodiversity, Food webs

## Abstract

Seagrass meadows play a key ecological role as nursery and feeding grounds for multiple fish species. Underwater Visual Census (UVC) has been historically used as the non-extractive method to characterize seagrass fish communities, however, less intrusive methodologies such as Remote Underwater Video (RUV) are gaining interest and could be particularly useful for seagrass habitats, where juvenile fish camouflage among the vegetation and could easily hide or flee from divers. Here we compared the performance of UVC and RUV methodologies in assessing the fish communities of two seagrass meadows with low and high canopy density. We found that RUV detected more species and fish individuals than UVC, particularly on the habitat with higher seagrass density, which sheltered more juveniles, especially herbivorous, and adult piscivorous of commercial importance, evidencing significant differences in energy flow from macrophytes to predators between seagrass habitats, and also differences in the ecosystem services they can provide. Considering the ongoing worldwide degradation of seagrass ecosystems, our results strongly suggest that fish surveys using RUV in ecologic and fisheries programs would render more accurate information and would be more adequate to inform the conservation planning of seagrass meadows around the world.

## Introduction

Seagrass meadows provide shelter and food resources that sustain high levels of marine biodiversity in complex trophic chains interconnected with adjacent ecosystems such as mangroves and coral reefs^1–5^. Seagrass attain some of the highest rates of primary production in aquatic environments^6^, helping to maintain good water quality by absorbing excess of nutrients and by trapping and stabilizing sediments^7–9^. Iconic organisms such as marine turtles and sirenians (manatees and dugongs) are highly dependent on seagrass habitats^10^. Unfortunately, the importance of seagrass meadows has been particularly overlooked in tropical environments, where the attention is focused on the colorful coral reef communities^11^. Seagrass ecosystems provide many goods and services to the human societies^12–14^. Among these, their fundamental role as nursery and feeding grounds for fish of ecological and commercial importance is highly valuable^15–20^. However, seagrass meadows are severely affected by multiple anthropogenic disturbances^21,22^, including heavy exploitation of their fishery resources^17,18,23^.

This demands urgent conservation planning, and a better understanding of the specific role of seagrass fish communities, as part of a highly interconnected net of marine organisms and ecosystems^11,24,25^. Indiscriminate extractive methodologies using different types of nets, such as throw traps, drop samplers, seine and trawling nets (Supplementary Table S1) are inappropriate for ecological monitoring programs, especially when sampling threatened or endangered species and on no-take marine sanctuaries^26^. The most common non-destructive method is Underwater Visual Census (UVC), as it is a low-cost technique, which allows fast data gathering (Supplementary Table S1). However, this method presents several disadvantages, as it strongly depends on the surveyor skills, water clarity and it is also affected by the diver presence, which incites many fish to flee or hide before being recorded^27–29^. Many of these problems can be overcome by using video in marine science^30^.

The development of video-sampling methodologies to study marine communities dates back from the 50 s, although it is not until recently that its use has spiked^30^, when video cameras are more affordable. The simplest of its kind, Remote Underwater Video (RUV), consists on a platform holding a battery-operated video camera inside a waterproof housing. Nowadays, action cameras offer a perfect balance between price, image quality, operability and inconspicuousness^31^. This approach allows recording less disturbed fish communities, reducing the bias in the assessment of community structure. Moreover, RUV has fewer limitations of time and depth, facilitating replication at wide spatial scales. The number of replicates obtained at the same time will depend on the number of video units in possession^32^. Following standardized procedures, RUVs can be used on different aquatic habitats^25^, minimizing the comparative problems presented by other approaches^33^. In addition, videos are permanent records, allowing future comparative analyses and can be used for campaigns of public awareness^30^.

The application of video-based methodologies is almost restricted to Oceania, USA and Europe, with marine biodiversity hotspots such as the Coral Triangle and the Caribbean Sea lagging behind^30^. The literature review we performed showed that only 13 studies have used video samplings to assess seagrass fish communities (Supplementary Table S1). Only 2% of studies in the world have used baited RUVs (BRUVS), relative to 43% of studies on rocky and coral reefs^34^, despite that RUV could be particularly useful in seagrass meadows, as many fish are wary juveniles that tend to flee in the presence of the diver and are also very good in camouflaging among the vegetation. These characteristics could reduce the effectiveness of UVC surveys. However, no comparison between both methodologies has been yet performed. Here, we aimed to address this knowledge gap by comparing the performance of RUV and UVC methods to assess: (i) species diversity and composition, (ii) abundance of different fish life-stages; and (iii) trophic guilds of seagrass fish communities. We selected two types of tropical seagrass meadows characterized by contrasting canopy morphology and density of the main habitat-builder *Thalassia testudinum: Backreef*, characterized by shorter leaves, lower Leaf Area Index (LAI) and above-ground seagrass biomass; and *Lagoon*, having longer leaves, larger LAI and higher above-ground biomass. Both habitats were previously described^35^.

## Results

### Species Richness

**Figure 1:**
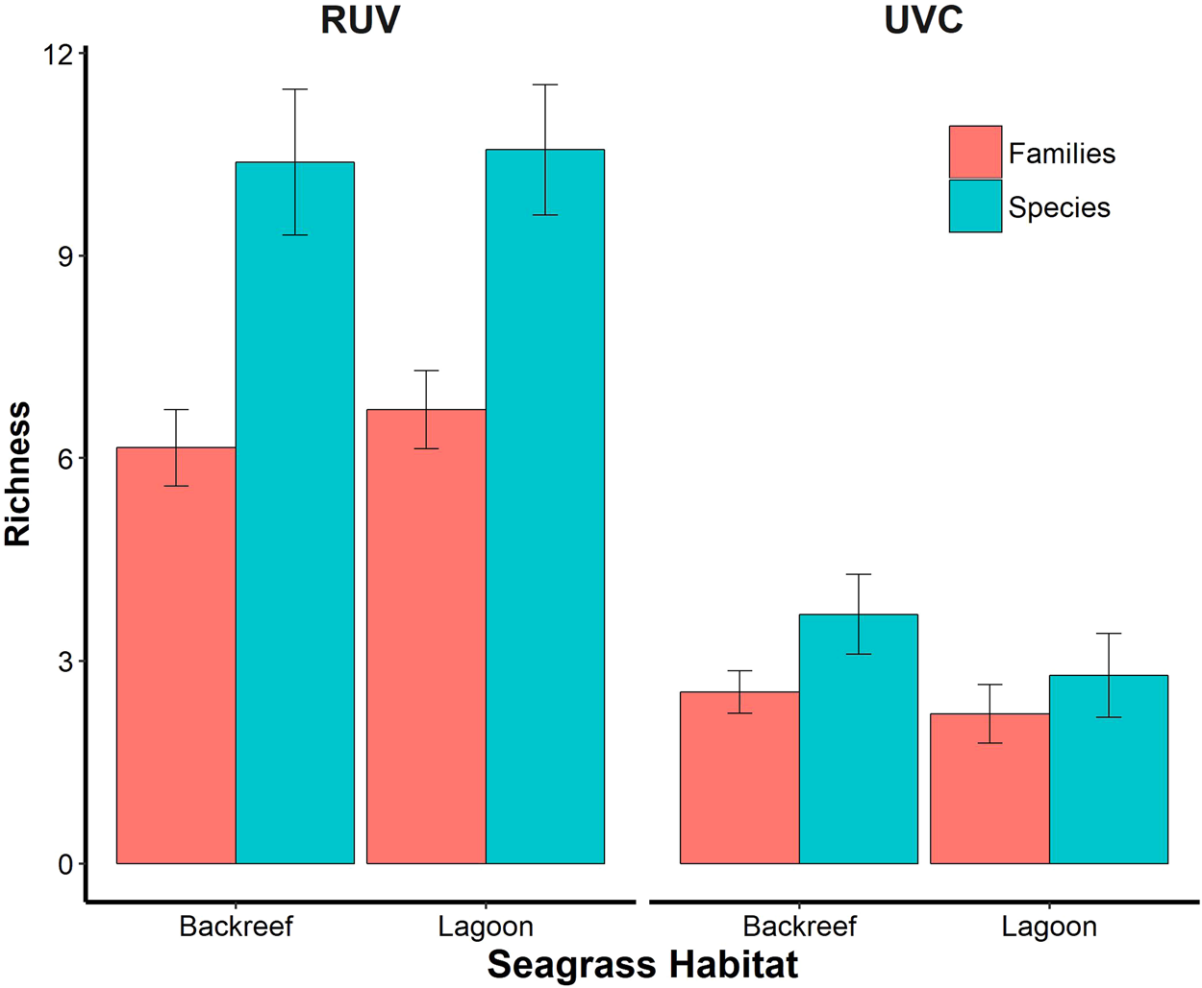
Number of fish species and families (mean ± se) registered by Remote Underwater Video (RUV) and Underwater Visual Census (UVC) for two seagrass meadows, Backreef and Lagoon, located in the reef lagoons of Cancun-Puerto Morelos (Mexico).

Both methods found higher species diversity in the Lagoon but RUV detected higher numbers of species and families on both habitats. We identified a total of 63 fish species belonging to 26 families, with RUV recording 48 species of 25 families and UVC registering 31 species of 17 families in total. RUV found 38 species (22 families) at the Lagoon and 34 species (18 families) at the Backreef, with 24 species (15 families) in common, whereas UVC registered 19 species (14 families) in the Lagoon and 16 species (9 families) at the Backreef, with only 4 species (6 families) found in both meadows. There were no significant differences in average species and family richness between habitats for both methods. However, differences between methods were significant in each habitat (Fig. 1), regarding fish families (PERMANOVA; Backreef: pseudo-F_1, 24_ = 31.408, *P* = 0.0001; Lagoon: pseudo-F_1, 26_ = 38.707, *P* = 0.0001) and species (PERMANOVA; Backreef: pseudo-F_1, 24_ = 29.375, *P* = 0.0001; Lagoon: pseudo-F_1, 24_ = 46.009, *P* = 0.0001).

Most of the species identified were of commercial importance for the fisheries or aquarium industries. In total, both methods detected 39 species with value for recreational and commercial fishing and 45 species categorized as valuable for private or public aquariums (Supplementary Table S2). RUV recorded 82% of fisheries species and 77.7% of aquarium species, while UVC registered 35.9% of fisheries species and 57.7% aquarium species. Among all the species, eight were cataloged as associated with seagrass habitats, RUV recorded all of them, while UVC only registered 37.5%. Species identified with transient behavior between coral reefs and seagrass meadows were 43, RUV recorded 76% of them and UVC registered 51.1%. Lastly, 12 species were categorized as reef residents during adulthood, RUV recorded 66.6% of these, while UVC registered 36.3% (Supplementary Table S2).

### Species Accumulation Curves

**Figure 2:**
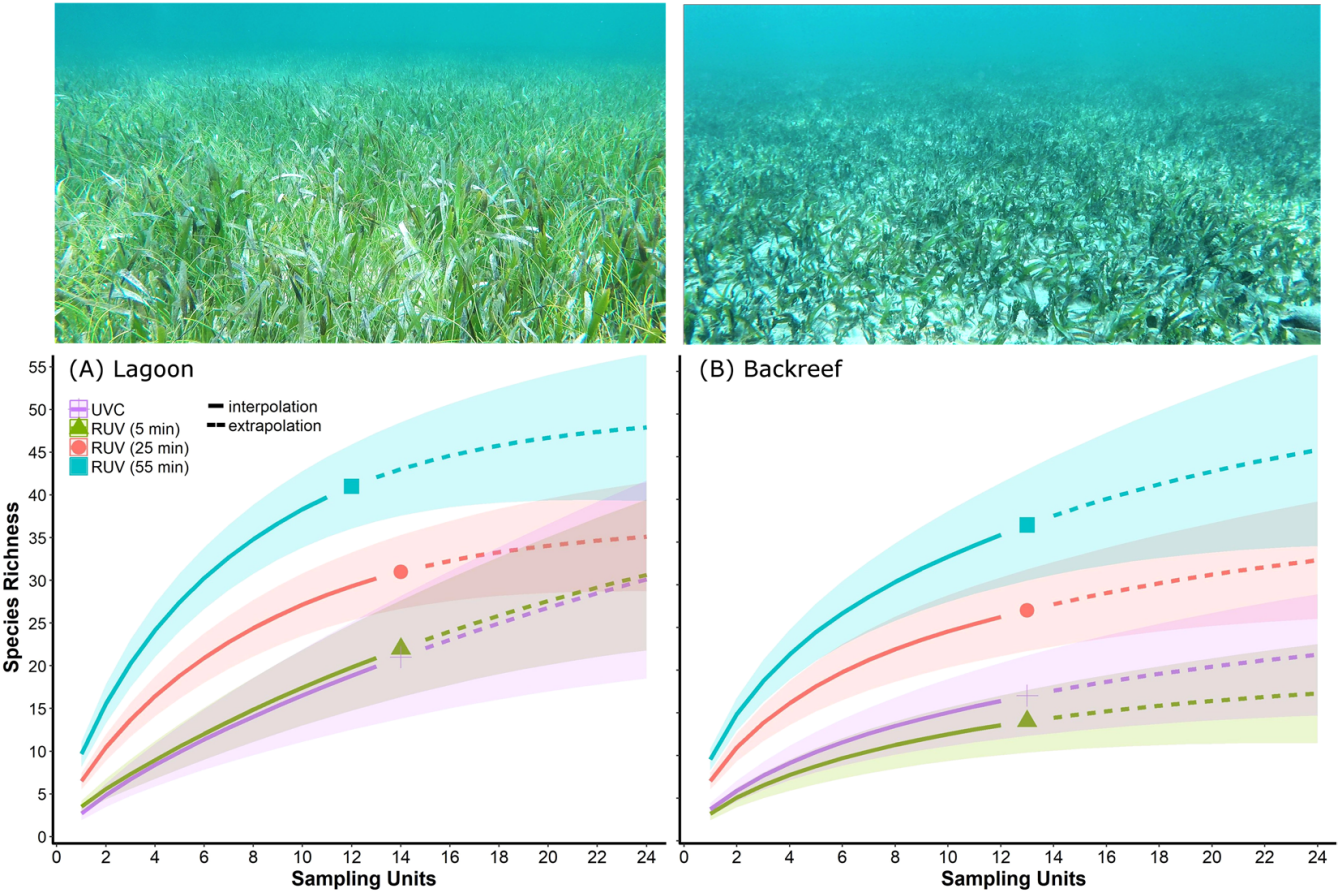
Fish species accumulation curves (estimates ± 95% confidence intervals) for Remote Underwater Video (RUV) at different recording length-times and Underwater Visual Census (UVC) for two seagrass habitats located in Cancun-Puerto Morelos (Mexico): Lagoon (**a**) and Backreef (**b**). Interpolation (solid lines) and extrapolation (dotted lines) curves were generated applying methodologies of Chao *et al*. (2004) using the package iNEXT of the R software (R Foundation).

RUV samples of 5 minutes registered similar species (22) than UVC (21) at the Lagoon after 14 sampling units and was predicted to reach 30 ± 9.8 species if sampling effort was increased to 24 RUVs, close to the prediction for UVC: 30 ± 11.7 (Fig. 2a). However, higher recording times in RUVs resulted in higher species richness. RUVs of 25 minutes registered 31 species during the observed surveys and would reach 35 ± 5.6 after 24 samples, while RUVs of 55 minutes yielded the maximum values of observed species richness (41) with less sampling units (n = 12) and would detect 46 ± 6.4 species after 19 samples (Fig. 2a). Species accumulation curves for the Backreef meadow presented similar patterns as described before, but with lower species richness. RUVs of 5 min and UVC again had similar values of species richness for observed (14 and 16 species respectively) and predicted surveys (n = 24; 17 ± 5.0 and 22 ± 8.1 respectively; Fig. 2b). However, RUVs of 25 minutes detected 27 species and would reach 33 ± 7.1 after 24 samples while RUVs of 55 minutes observed 46 species and would record 46 ± 10.3 species after 25 samples (Fig. 2b). Further sampling estimations indicate that UVC surveys would require about 50 samples in the Lagoon and 60 samplings at the Backreef to detect similar richness values than RUVs of 45 minutes.

### Abundance and Species Composition

**Figure 3:**
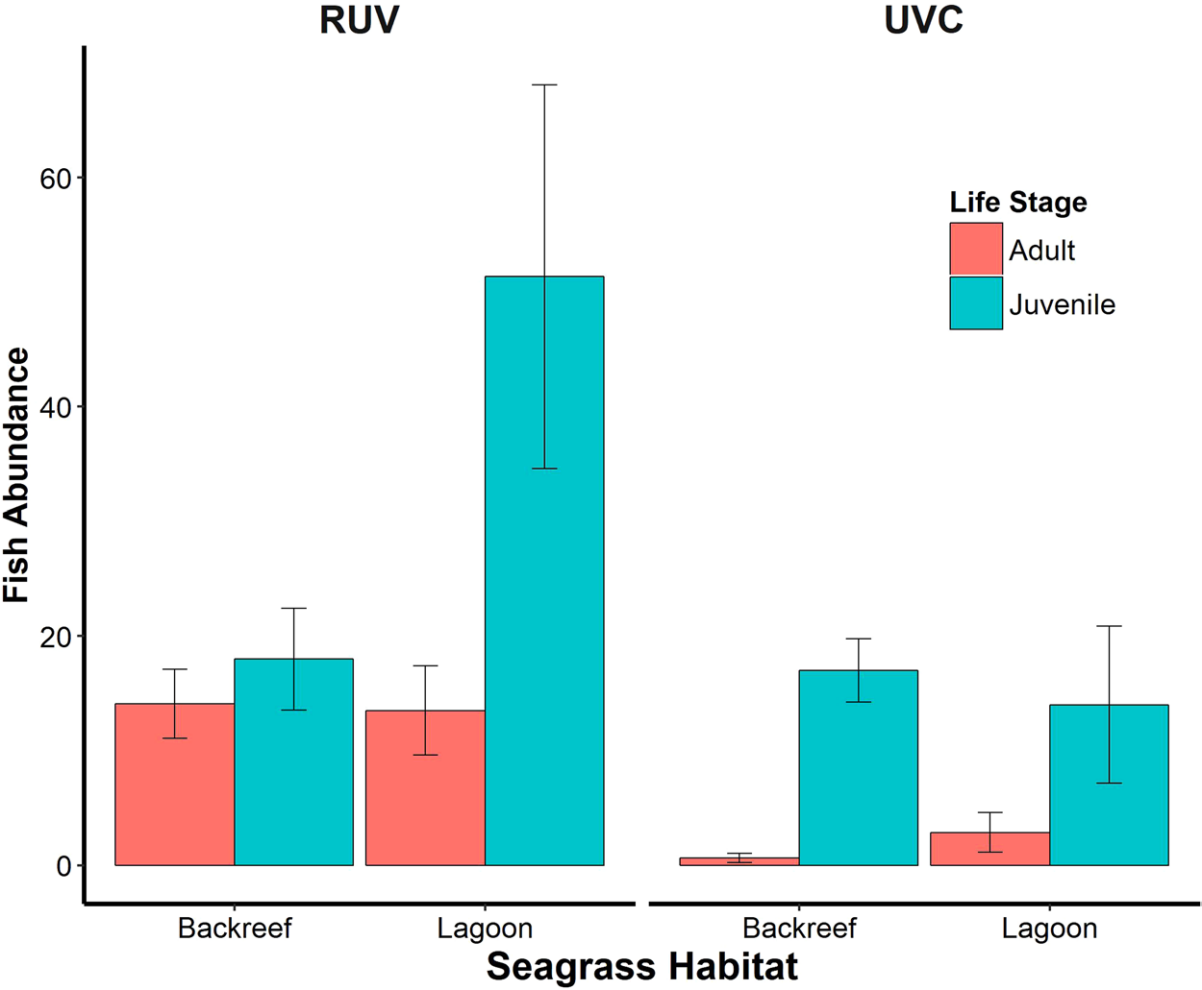
Fish abundance (mean ± se) by life stage registered by Remote Underwater Video (RUV) and Underwater Visual Census (UVC) at two seagrass habitats located in the reef lagoon of Cancun-Puerto Morelos (Mexico): Backreef and Lagoon.

The fish communities characterized by both methods also differed in fish abundance. RUV registered significantly more individuals of all life stages than UVC in both seagrass meadows with the exception of juveniles at the Backreef (Fig. 3; Table 1). UVC detected a total of 232 individuals in the Backreef, of which 95% were juveniles, and 236 individuals in the Lagoon (95% juveniles). On the other hand, RUV recorded 427 individuals in the Backreef (55% juveniles) and 930 in the Lagoon (73% juveniles). Scarids accounted for most of the differences in juvenile abundance between methods. For UVC, scarids accounted for 11% (22 juveniles) in the Lagoon and 5% (12 juveniles) in the Backreef, while for RUV, scarids accounted for 48% (330 juveniles) of the juveniles in the Lagoon and 58% (136) in the Backreef. Both methodologies did not detect significant differences in adult fish abundance between habitats (UVC: Lagoon: 2.86 ± 1.73 and Backreef: 0.61 ± 0.40; pseudo-F1, 25 = 1.7844, *P* = 0.2376). However, RUV recorded higher abundances than UVC (RUV: Backreef: 14.07 ± 3.0 and Lagoon: 13.50 ± 3.89; PERMANOVA; pseudo-F1, 25 = 0.017473, *P* = 0.7835). For juveniles UVC found slightly higher abundances at the Backreef (Lagoon: 14.0 ± 6.85; Backreef: 17.0 ± 2.77; PERMANOVA; pseudo-F1, 25 = 3.0124, *P* = 0.0239). In contrast, RUV’s data showed that the Lagoon sheltered higher amounts of juvenile fish (Fig. 3; Backreef: 18 ± 4.45 and Lagoon: 51.36 ± 16.75; PERMANOVA; pseudo-F1, 25 = 2.0353, *P* = 0.1496).

**Table 1:** PERMANOVA results testing for differences between Remote Underwater Video and Underwater Visual Census in abundance of juvenile and adult fish inhabiting two seagrass meadows located in the reef lagoons of Cancún- Puerto Morelos (Mexico) reef lagoon (Cancun, Mexico): Backreef and Lagoon

Habitat	Life stage	Pseudo-F	*P*-value
Backreef	Juveniles Adults	F_1, 24_ = 0.13473 F_1, 24_ = 34.003	0.8085 **0.0001**
Lagoon	Juveniles Adults	F_1, 26_ = 3.3828 F_1, 26_ = 12.932	**0.0084** **0.0001**

**Figure 4:**
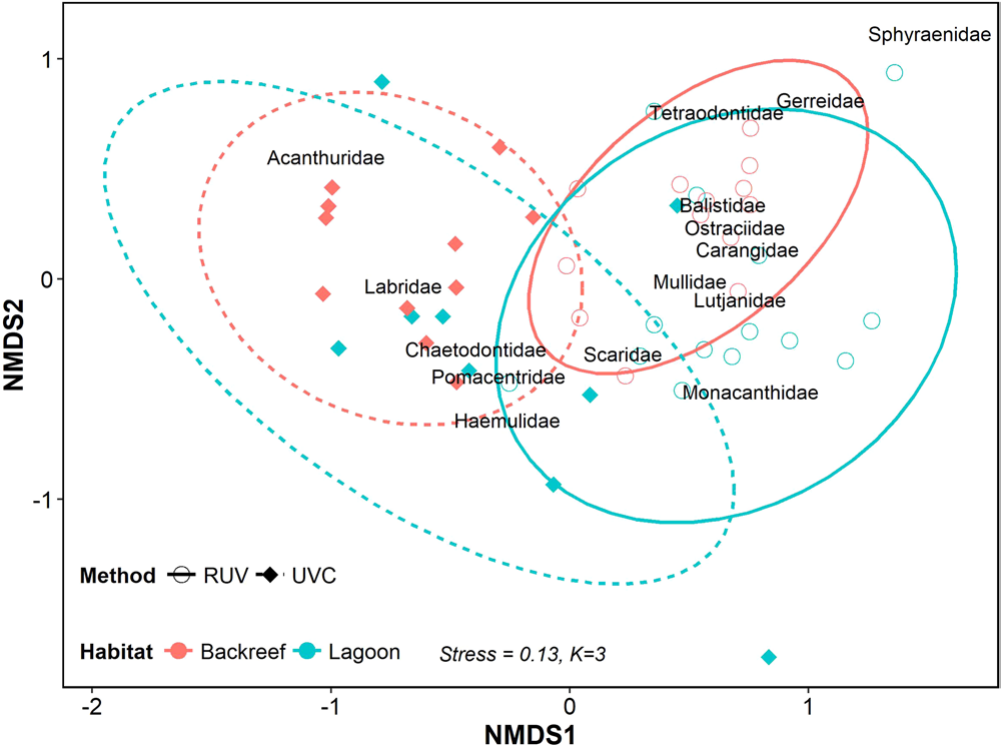
Non-metric Multidimensional Scaling of the fish communities registered by Remote Underwater Video (RUV) and Underwater Visual Census (UVC) at two seagrass habitats located in the reef lagoons of Cancun-Puerto Morelos (Mexico): Backreef (red) and Lagoon (blue).

The differences in fish abundance between methods were driven by different species. The fish communities inhabiting seagrass with low and high canopy density were different according to surveys with UVC (ANOSIM, *P* = 0.001) and RUV (ANOSIM, *P* = 0.005), however, each methodology registered different species assemblages (ANOSIM, *P* = 0.0001). Species of the families *Acanthuridae*, *Labridae*, *Chaetodontidae*, *Pomacentridae* and *Haemulidae* were preferentially detected by UVC, whereas species of the families *Sphyraenidae*, *Gerreidae*, *Tetraodontidae*, *Balistidae*, *Ostraciidae*, *Carangidae*, *Mullidae*, *Lutjanidae*, *Scaridae* and *Monacanthidae* were predominantly detected by RUV (Fig. 4; Supplementary Table S2).

**Figure 5:**
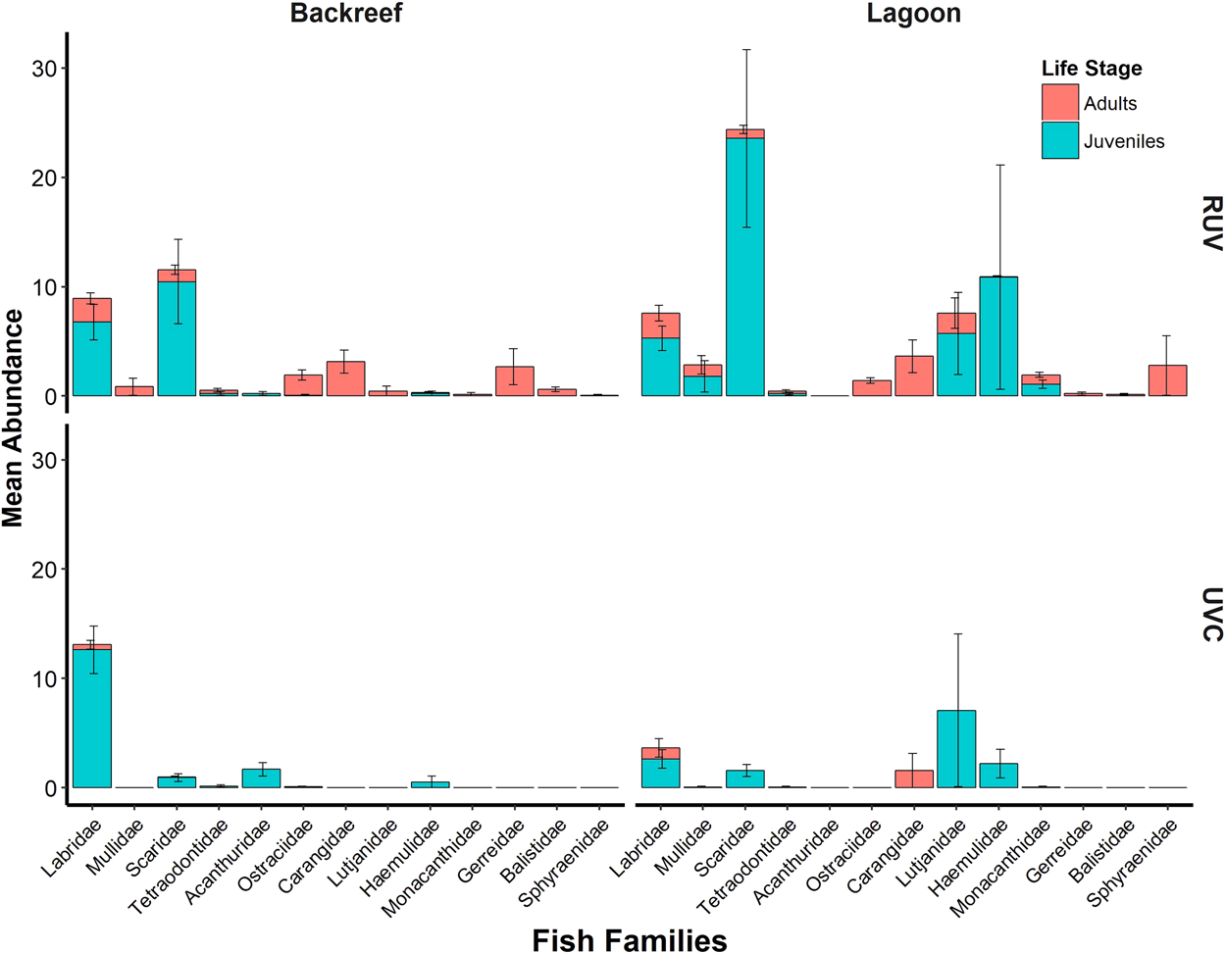
Fish abundance (mean ± se) by life stage of the most important fish families registered by Remote Underwater Video (RUV) and Underwater Visual Census (UVC) at two seagrass habitats located in the reef lagoons of Cancun-Puerto Morelos (Mexico): Backreef and Lagoon.

Surveys with UVC at the Backreef registered many labrids in juvenile stage (12.61 ± 2.19), seconded by acanthurids (1.69 ± 0.61), scarids (0.92 ± 0.35) and haemulids (0.53 ± 0.53), while among the adult fish registered the most abundant were labrids as well (0.46 ± 0.38). At the Lagoon, juvenile fish were mostly lutjanids (7.07 ± 6.99), labrids (2.64 ± 0.84), haemulids (2.21 ± 1.30), and scarids (1.57 ± 0.56), while the most abundant adult fish were carangids (1.57 ± 1.57) and labrids (1 ± 0.85) (Fig. 5; Supplementary Table S2). In comparison, RUV surveys at the Backreef detected many juvenile scarids (10.46 ± 3.86), labrids (6.76 ± 1.62), haemulids (0.23 ± 0.23), tetraodontids (0.23 ± 0.16) and acanthurids (0.23 ± 0.16), while the most abundant adult fish were carangids (3.15 ± 1.04), gerreids (2.69 ± 1.64), labrids (2.15 ± 0.50), ostraciids (1.84 ± 0.46), scarids (1.07 ± 0.43), mullids (0.84 ± 0.76), balistids (0.61 ± 0.21), lutjanids (0.46 ± 0.46), dasyatids (0.38 ± 0.14) and tetraodontids (0.30 ± 0.17). At the Lagoon, juvenile scarids presented the higher abundances (23.57 ± 8.12), seconded by haemulids (10.85 ± 10.24), lutjanids (5.71 ± 3.75), labrids (5.28 ± 1.11), mullids (1.78 ± 1.4), and monacanthids (1.07 ± 0.38), while adult fish were mostly carangids (3.64 ± 1.50), sphyraenids (2.78 ± 2.71), labrids (2.28 ± 0.71), lutjanids (1.85 ± 1.38), ostraciids (1.42 ± 0.25), mullids (1.07 ± 0.85), monacanthids (0.85 ± 0.23), scarids (0.78 ± 0.38) and urobatids (0.28 ± 0.16) (Fig. 5; Supplementary Table S2). Many juvenile scarids were not possible to be identified to species (463 individuals) due to their similarity in early stages and their capacity to change coloration in drastic ways^36^

### Trophic groups

**Figure 6:**
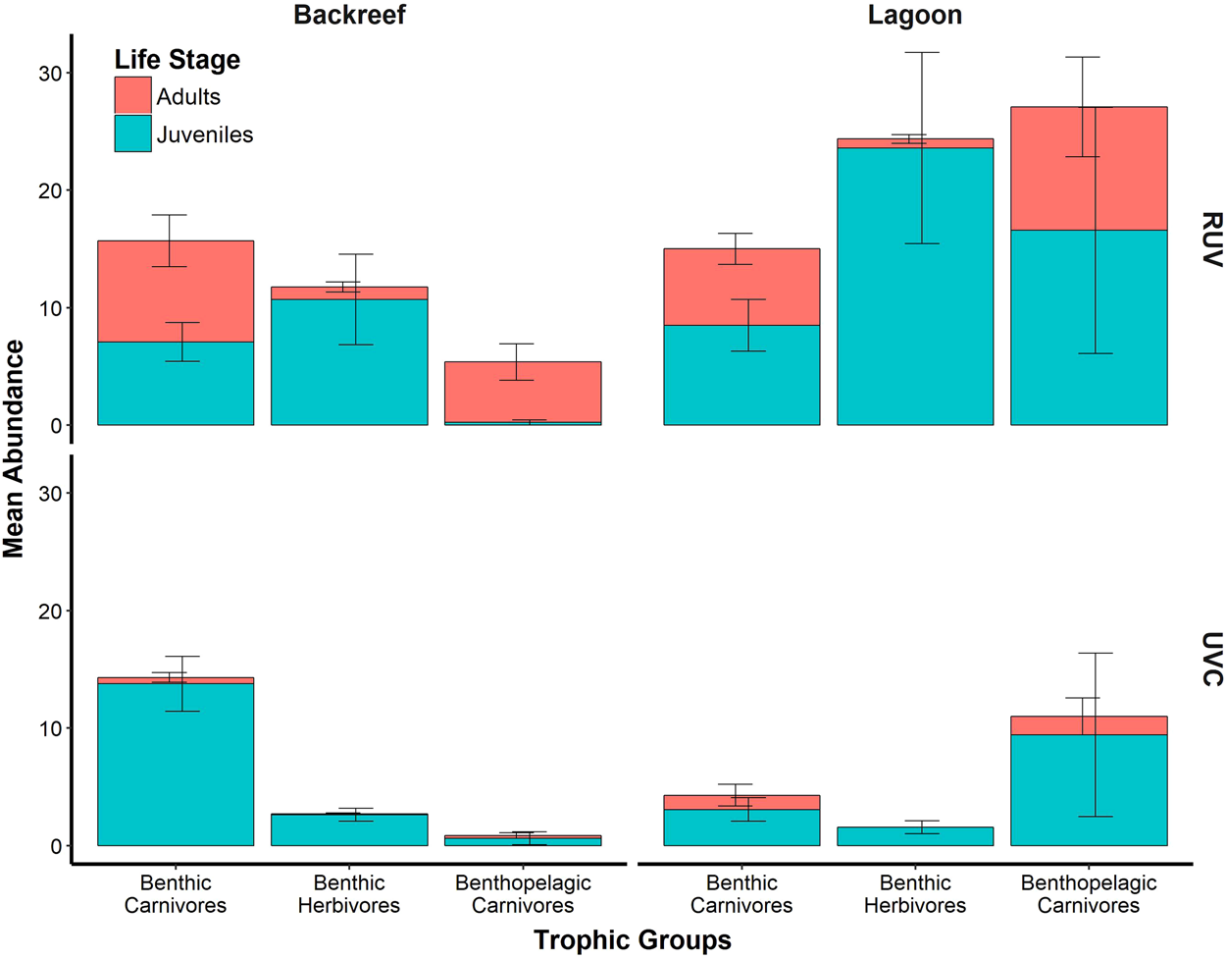
Fish abundance (mean ± se) by life stage of the general trophic groups registered by Remote Underwater Video (RUV) and Underwater Visual Census (UVC) at two seagrass habitats located in the reef lagoons of Cancun-Puerto Morelos (Mexico): Backreef and Lagoon.

RUV captured higher abundances than UVC of almost all trophic groups in both seagrass habitats (Fig. 6; Table 2). Trophic information at the Backreef registered with UVC showed that benthic carnivores were more abundant (13.77 ± 2.34) than benthic herbivores (2.61 ± 0.55) and benthopelagic carnivores (0.61 ± 0.53; PERMANOVA, pseudo-F_1, 25_ = 18.64, *P* = 0.0004), while at the Lagoon benthopelagic carnivores were more abundant (9.42 ± 7.20) than benthic carnivores (3.07 ± 0.77) and herbivores (1.57 ± 0.58; PERMANOVA, pseudo-F_1, 25_ = 2.1024, *P* = 0.1106). Similarly to UVC, the surveys of RUV showed that benthic carnivores were the most abundant at the Backreef (15.69 ± 2.62; PERMANOVA, pseudo-F_1,25_ = 5.3968, *P* = 0.0028), however, the abundances of benthic herbivores (11.76 ± 3.81) and benthopelagic carnivores (5.38 ± 1.55) were higher. At the Lagoon, the pattern was different, with benthic herbivores (24.35 ± 8.10) and benthopelagic carnivores (25.5 ± 13.63) having higher abundances than benthic carnivores (15 ± 3.63; PERMANOVA, pseudo-F_1, 25_ = 2.3999, *P* = 0.0282) (Fig. 6).

**Table 2:** PERMANOVA results testing for differences between Remote Underwater Video and Underwater Visual Census in abundance of the main trophic groups inhabiting two seagrass meadows located in the reef lagoons of Cancun-Puerto Morelos (Mexico): Backreef and Lagoon.

Habitat	Trophic group	Pseudo-F	*P*-value
Backreef	Benthic carnivores	F_1, 24_ = 0.13694	0.8703
	Benthic herbivores	F_1, 24_ = 3.8988	**0.0034**
	Benthopelagic carnivores	F_1, 24_ = 33.116	**0.0001**
Lagoon	Benthic carnivores	F_1, 26_ = 5.8321	**0.0007**
	Benthic herbivores	F_1, 26_ = 6.8789	**0.0008**
	Benthopelagic carnivores	F_1, 26_ = 4.4279	**0.0124**

The patterns previously described for UVC were mainly based on juvenile individuals, since few adults were detected (Fig. 6). In contrast, RUV recordings allowed to distinguish patterns among different life-stages in low and high canopy density. Adult fish at the Backreef were mostly benthic (8.61 ± 2.20) and benthopelagic carnivores (5.15 ± 1.56), whereas juveniles were mostly benthic herbivores (10.69 ± 3.83) and carnivores (7.07 ± 1.64). At the Lagoon, adult fish were primarily benthopelagic (10.50 ± 4.24) and benthic carnivores (6.50 ± 1.31), whereas juveniles were mostly benthic herbivores (23.57 ± 8.12), followed by benthic (8.5 ± 2.19) and benthopelagic carnivores (16.57 ± 10.48) (Fig. 6). Additionally, RUV recorded other organisms aside from fish with important ecological roles, such as marine turtles (*Chelonia mydas*; Lagoon: 0.14 ± 0.09; Backreef: 0 ± 0) and schools of Caribbean squids (*Sepioteuthis sepioidea*; Lagoon: 1.64 ± 1.42; Backreef: 0.76 ± 0.56).

## Discussion

The comparison performed here highlighted significant differences between Underwater Visual Census (UVC) and Remote Underwater Video (RUV) in their effectiveness to characterize fish communities of tropical seagrass meadows at worldwide scale. RUV recorded higher species diversity and abundance of fish individuals of all life-stages and trophic groups than UVC, despite the fact that abundance data recorded by RUV (*i.e*. MaxN) is a conservative measure designed to avoid double counting, and thus represents the minimum abundance per species. Accordingly, it is still likely that we underestimated real abundances of fish populations with high densities^37^. Our analysis indicates that 55 minutes of recording with the RUV method approached the asymptote of the species richness curves much faster than lower recording times or UVC transects. This agrees with the literature in that recording for more than 30 minutes may be more cost-effective to assess fish diversity in coral reefs^38^ and seagrass meadows^39^. The ability of RUV to carry out higher sampling effort (as time) per location, combined with the absence of the disturbance associated with the presence of divers^40^, may explain its higher effectiveness for describing fish communities.

Past surveys on seagrass habitats on the northern Mexican Caribbean (Cancun and Puerto Morelos) registered 62 species of fish after 36 UVC transects of 50 meters^41^, while surveys in the southern region (Mahahual and Xcalak) found 28 species after 48 transects of 20 meters^42^. Although a direct comparison of our results with these previous studies is not possible due to spatial and temporal mismatches^43^, the values show a good approximation to the sampling effort predictions of our species accumulation curves, where UVC would require more than double the sample units to approximate the species richness found by RUVs of 55 minutes. These differences could be even higher, as we carried out UVC transects before setting up the RUV units, which might have caused some fish to flee. We considered five minutes of buffer between techniques, which is a common time frame used in comparative studies^40^. However, this might have not completely off-set the “diver-effect” in the RUV results^27^.

### Canopy structure

Previous studies have not documented such striking differences between diver and video-based sampling techniques regarding fish abundance and species richness in reef ecosystems^29,44^. However, both techniques have been found to differ on species composition, depending on the habitat and behavior of each species. The higher the topographic complexity of the reef habitat, the more effective UVC could be, since fish can avoid being recorded in videos by sheltering on caves, cracks or overhangs^44–47^. Species with wary behavior that tend to flee from divers have higher probability to be recorded by RUV and bold species such as site-attached fish, can be easily detected by UVC, while mobile conspicuous species, such as labrids, present similar likelihood to be detected by both methodologies^44^. Our results partially agreed with these past observations. However, the seagrass environment seemed to dwarf some of the benefits traditionally attributed to UVC, as we found that RUV characterized better the fish communities, particularly the habitat with higher canopy density. In agreement with previous studies UVC registered seven site-attached species of haemulids, chaetodontids, pomacentrids and sciaenids that were not recorded by RUV (Supplementary Table S2). This was due because UVC surveyed larger areas and more micro-habitats within the same meadow, such as exposed seagrass roots; empty snail shells of the “queen conch” *Lobatus gigas*, octocorals growing on rocks, or coral bummies^48^. When these microhabitats where sporadically seen within the RUVs field of view, the species sheltering within, such as acanthurids, were recorded. However, most of the time the cameras were oriented toward the seagrass canopy, where RUV outperformed UVC. Unlike other studies using horizontal RUVs, we set up our video-camera system slightly facing down precisely to target cryptic individuals. For example, 30 species were exclusively detected by video, many of which were inconspicuous individuals sheltering among the seagrass leaves, such as solitary monacanthids, tetraodontids and labrids and schooling juvenile fish roaming among or above the canopy, like scarids, mullids and lutjanids, in addition to adult carangids, gerreids and sphyraenids (Supplementary Table S2). Nonetheless, future RUV deployments must consider the spatial distribution of all microhabitats within a particular seagrass bed to guarantee their inclusion in the surveys.

Only RUV recorded higher abundance of individuals and species in juvenile stage at the more dense seagrass canopy of our study, supporting previous studies reporting positive correlations between the density of the dominant seagrass and species richness and abundance of fish^4,49–53^. Explanations for this suggest that denser and taller seagrass mass provides more physical protection and shade^54^ to juvenile fish. Indeed, seagrass meadows with higher LAI present higher light attenuation and leaf self-shading within the canopy^35^, which allows juvenile fish to hide. The fact that RUV registered hundreds of juvenile individuals more than UVC in the Lagoon, but not at the Backreef, supports that the denser the seagrass canopy, the more protection it provides to juvenile fishes. This finding also highlighted a negative effect of canopy density on the capacity of UVC to detect fish individuals, which explains why this method was less efficient to evaluate the ecological value of this particular meadow in comparison with RUV. We may conceptualize this by considering dense seagrass canopies as a curtain that cannot be uncovered without disturbing the community, in contrast with coral reefs where divers can easily look into cracks, caves or overhangs and detect the fish hiding within. The species accumulation curves may reflect this, as the curve for UVC estimated for the Lagoon showed a steeper slope than that of the Backreef. This means that the description of the potential fish diversity in seagrass habitats with higher canopy density requires more sampling effort. This seemed to be particularly important for scarids, which accounted for most of the differences in juvenile abundance. The ecological importance of this group in marine ecosystems makes these results highly relevant at global scale for the development of monitoring programs on seagrass meadows^55^ and on adjacent ecosystems^56^.

### Predatory fish

Fish surveys using video techniques in reef systems have registered more adult predators than diver-based methods, this could be attributed to the addition of bait (*e.g*. pilchard) on RUV systems^44,45,57,58^. In our study we didn’t use bait, but still found that RUV recorded more adult individuals than UVC, particularly more carnivores valued in the fishing industry. Indeed, RUV recorded higher number of species and individuals in adult stage, describing more accurately trophic interactions in the ecosystem, as the few adult organisms registered by UVC surveys depicted incomplete trophic patterns. The use of bait in RUV sampling reefs has been an effective way to attract more individuals and species to the video cameras^45,58,59^. However, its use in seagrass meadows may not be so positive, as we observed in the videos that juvenile fish rushed for shelter when carnivorous fish such as *Caranx ruber* were swimming close by. As multiple predators may remain permanently around the camera in the presence of bait, many juvenile fish would be scared away, impacting the estimations of abundance and species richness.

The trophic patterns documented seem to be related to the availability of food resources in each seagrass habitat, as previously reported^60,61^. Food resources can be as determinant for fish abundance and community structure as refuge availability^62^. Differences between habitats were primarily reflected in higher abundance of herbivorous and piscivorous in the Lagoon. Higher biomass of palatable epiphytes available on a larger leaf area (higher LAI) could explain the higher number of juvenile herbivorous^63,64^. At the same time, the higher abundance of juveniles may attract piscivorous species from nearby habitats^65^. However, the protection provided by the more dense seagrass canopy may reduce mortality rates, maintaining high abundances of juveniles at the Lagoon^66^. In contrast, benthic carnivores were dominant in the Backreef, although at the same abundance than in the Lagoon, suggesting that they could feed equally on both habitats. Ostracids were observed roaming and feeding in both habitats, but we just saw dasyatids digging and ingesting sediments at the Backreef. This suggests that less dense canopies may facilitate access to the sediment, making easier to dig out invertebrates, while denser canopies may provide more food among and/or on the leaves. The patterns of abundance, species richness and trophic groups documented here are likely to change in space and time^67^ associated with diurnal changes^68^ and seasonal fluctuations in water temperature and irradiance. These factors determine the reproduction timing of many fish species, as well as changes in canopy structure and in the primary production of the seagrass habitat, which in turn affects its shelter capacity and the amount of food resources available^69^.

An additional point to be considered is the capacity of RUV methodologies to determine fish sizes, which can be used to calculate biomass through known length-weight relationships^37^. Sizes can be estimated by using calibration scales^46^, using parallel lasers^70^ or using allometric relationships, such as eye to head-height proportions^71^. The limitation of all these approaches is that not all individuals sighted can be sized. The mean error using calibration scales was estimated as 14.3 ± 2 mm^72^ and has been applied successfully in large scale ecological studies^73,74^. In contrast, fish sizes are routinely estimated in almost all the fish while performing UVC. However the accuracy of these estimations are highly variable and dependent on the skills of the diver, the environmental conditions and the size of the fish. UVC accuracy errors have been estimated to be −20.1 ± 0.6 mm under controlled pool conditions^75^, while in the natural environment divers tended to underestimate the length of small fish (175 mm) by 35 mm and overestimate big fish (400 mm) by 40 mm^76^. Stereo-video systems have been developed to overcome these biases and can successfully measure length with minimal error (0.2 ± 0.4 mm)^72^. The drawback of this technology is that the equipment for sampling, storage information and the required software for video analyses increase the costs of the survey program considerably. However, as these technologies advance and their use increases, their accessibility is also enhanced^77^. An example can be found in Cuba, where researchers have been able to operate stereo-video technologies since 2011^78^.

## Conclusions

As the degradation of seagrass meadows progresses around the world, the need to plan and support conservation actions becomes urgent, therefore accurate environmental and biological information is needed. Our study demonstrates that Remote Underwater Video (RUV) is a more effective tool than Underwater Visual Census (UVC) for describing the fish communities of seagrass meadows. The finding suggests that past descriptions relying on UVC surveys could have underestimated the abundance and species diversity of seagrass habitats, especially under limited sampling efforts. The fact that our analysis was performed in a tropical environment with high levels of fish diversity supports the utility of video technologies to sample in other regions with lower diversity such as temperate habitats. The use of a common methodology in different parts of the world is fundamental for comparative studies. For these worldwide comparative purposes, the use of techniques that carry less bias is particularly recommendable. The results obtained using RUV surveys highlighted the importance of the density of the seagrass canopy for structuring and maintaining the fish communities of marine coastal ecosystems. By extension, these results are also relevant to preserve those communities with particular value for recreational and commercial fisheries. Our results indicate that changes in seagrass abundance, but in particular the loss of seagrass cover will severely impact fish abundance and biodiversity, in addition to other losses in marine resources and ecosystem services. This study therefore strongly recommends RUV methods for fish surveys within ecological, conservation and fisheries monitoring programs carried out on seagrass meadows. We especially encourage its use in tropical regions, where the application of video-technologies is still lagging behind, despite the higher biodiversity and inter-connectivity of seagrass meadows with other important ecosystems such as coral reefs and mangroves.

## Methods

### Sampling Design

The study was carried out in the Mexican Caribbean within Puerto Morelos National Park, located at the northernmost section of the Mesoamerican Barrier Reef. This marine system presents fringing reefs located about 1 to 2 km away from the coast. The reefs buffer the energy of the waves and allow the development of extensive inshore reef lagoons, 4–5 m of maximum depth, where seagrass meadows develop^35,79^. The morphotype of the dominant seagrass *Thalassia testudinum* changes significantly within the lagoon (see Enríquez and Pantoja-Reyes^35^). In the middle, where the sandy sediment is deeper, seagrass above-ground biomass and canopy height are higher. However, as the seagrass approaches the back-reef hard substrate is more present and the sediment becomes thinner. Seagrass meadows nearby the back-reef have reduced above-ground biomass and the canopy becomes less dense and with shorter leaves^35^.

We characterized here the fish communities inhabiting seagrass habitats with low (Backreef) and high canopy density (Lagoon) at three sites (Nizuc, Limones and Puerto Morelos) located in the northern Mesoamerican Barrier Reef System (from Cancun to Puerto Morelos). In each sampling point we performed underwater visual census (UVC) along 50 × 3 m transects (7 ± 1.2 min), a common sampling area in seagrass surveys (Supplementary Table S1). In total, 14 transects were surveyed at the Lagoon and 13 at the Backreef (4–5 per habitat in each site). The UVC surveyor registered all the fish individuals at sight by species and ontogenetic stage (juvenile or adult) based on morphometric features. After the UVC ended, a RUV system consisting on a GoPro camera attached to a frame elevated 50 cm above the ground was set-up at the middle point of the UVC transect (*i.e*. same number of sampling units than UVC). To enhance the detection of juvenile and cryptic species our video camera was set horizontally but slightly facing downwards so that the substrate covered ¾ and the water column ¼ of the video frame. No bait was used (*e.g*. BRUV), as we were interested on the effect of habitat structure to the fish community and bait releases produce an odor plume that can extend for hundreds of meters, attracting carnivorous fish from adjacent areas and disrupting the original community at the sampling point. Videos were recorded at a resolution of 2.7 K with medium field of view and 60 frames per second. We retrieved the video cameras after 1 hour, a common recording time (Supplementary Table S1), and calculated species accumulation curves at different time intervals (5, 25, 55 minutes) to assess optimal sampling times. The first five minutes of the video were not analyzed to exclude any disturbance that UVC might have created. Sampling points were located at least 500 meter apart from each other and fish abundance from RUVs were calculated as MaxN, the maximum number of individuals of a species in a single frame, a conservative index of abundance that avoids double counting fish^30^. In occasions, we added individuals from more than one frame when organisms of different ontogenetic stages of the same species were clearly distinguishable while they were registered. Species were classified as juveniles and adults (including initial phase of scarids and labrids) and in three main groups of trophic level and residency behavior combined for simplicity: benthic herbivores (diet based on macrophytes), benthic carnivores (diet based on invertebrates and plankton, including benthic omnivores) and benthopelagic carnivores (diet based on nekton, including pelagic carnivores). Fish species identifications and categorization of life-stage, trophic group, habitat associations and commercial importance were based on the field guides^80,81^ and information available on FishBase^82^.

### Statistical Analysis

Statistical differences in families and species richness and total abundance (number of individuals) by different life-stages between methods (UVC and RUV) and seagrass habitats (Backreef and Lagoon) were statistically tested with two-way Permutational Multivariate Analysis of Variance (PERMANOVA) with Method and Habitat as fixed factors and Site as random factor, followed by pairwise comparisons^45^. Differences in fish community composition between methods and habitats were analyzed with Non-metric Multidimensional Scaling (nmMDS) and Analysis of Similarity (ANOSIM) to test for statistical significance. PERMANOVA Tests were performed with the software PRIMER 6 & PERMANOVA + (PRIMER-E Ltd) and metaMDS and anosim for ordination analyses were done with the program R (The R project) using the package Vegan^83^. Abundance data was square root transformed to reduce the effect of schooling species. All tests were based on 9999 permutations and resemblance measures from bray curtis distances.

Species accumulation curves were generated for each habitat and sampling methodology, considering different recording length-times for RUV. For this, we used the package iNEXT (iNterpolation and EXTrapolation) of the software R, which uses rarefaction to calculate species accumulation and sample completeness curves based on the species presence/absence data and predict new species detection with future sampling effort; it also generates confidence intervals for the curves (±95%) by bootstrapping^84^.

### Equipment and Settings for Figures

All graphics in the manuscript were generated with the package ggplot of the software R (The R project). Panel labels and photographs were added using the graphics software GIMP 2. Photographs in Fig. 2 were captured by Z.P. with a camera GoPro Hero Black 4.

## Supplementary information

Supplementary tables

## Data Availability

All data generated in this study are available after request.
