# Supplementary material for: Remote underwater video reveals higher fish diversity and abundance in seagrass meadows, and habitat differences in trophic interactions
Source: Sci Rep. 2019 Apr 29;9:6596. doi: 10.1038/s41598-019-43037-5 (PMC6488625; doi:10.1038/s41598-019-43037-5)
Supplement: Supplementary file 1 — Supplementary tables [file 41598_2019_43037_MOESM1_ESM.pdf]

Remote underwater video reveals higher fish diversity and abundance in seagrass meadows, and habitat differences in trophic interactions

Authors: Salvador Zarco-Perello & Susana Enríquez

Supplementary Table S1. Studies of seagrass fish communities around the world using different methodologies. The list of studies using video technologies is exhaustive at the moment of submission (R-RUV: Rotating Remote Underwater Vehicle; S-RUV; Stereo-RUV; BRUV: Baited-RUV; S-BRUV: Stereo-BRUV; S-TOWV: Stereo Towed Video), studies using Underwater Visual Census (UVC; Tr: transect. Qd: quadrat.) and nets are just a sample among all the published studies in the literature. Seagrass+ indicates the inclusion of more habitats other than seagrass, Fish+ indicates if the study considered other taxa apart from fish and Juv+ indicates the inclusion of juveniles and adult fish.

**Sampling Method/Analisis index**	**Samples/time of video**	**Habitat**	**Region**	**Ontogeny**	**Seagrass species**	**Fish Species Richness**	**Method Comparison**	**Reference**
RUV	No access to paper	Seagrass	Myanmar	No access	No access to paper	12	na	Jones et al. 2018
R-RUV	10 min<br>n= 221	Seagrass +	New Caledonia	Fish / Juv	-	149 (all habitats)	na	(Pelletier et al. 2012)
S-RUV	50 min<br>n= 6	Seagrass +	North Sea, Sweden	Fish / Juv+	*Zostera marina*	9	na	(Perry et al. 2018)
S-BRUV, S-TOWV	60 min<br>n=217	Seagrass +	South Western Australia	Fish / Adult	*Posidonia spp.*,<br>*Amphibolis spp.*,<br>*Halophila ovalis*	Limited to 6	Habitat associations: TOWV>BRUV	(Galaiduk et al. 2017)
S-BRUV	60 min<br>n=24	Seagrass +	South Western Australia	Adult	-	38	eDNA	Stat et al. 2018
S-BRUV	60 min<br>n= 188	Seagrass +	South Western Australia	Adult	-	19	na	(Harvey et al. 2013)
BRUV	30, 60, 90 min<br>n= 12	Seagrass +	NSW, Australia	Fish / Adult	*Posidonia australis*	29	na	(Gladstone et al. 2012)
BRUV / Time	20 min<br>n= 22	Seagrass +	Newfoundland, Canada	Fish / Juv+	*Zostera marina*	7	BRUV vs Seine	(Dalley et al. 2017)
BRUV / MaxN	60 min<br>n= 57	Seagrass	South Australia	Fish / Adult	*Posidonia spp.*,<br>*Halophila spp.*, *Heterozostera spp.*	36	na	(Whitmarsh et al. 2014)
S-BRUV / MaxN	55 min<br>n=11	Seagrass +	UK	Fish / Juv+	*Zostera marina*	Focused on gadoids	na	(Elliott et al. 2017)
S-BRUV/ MaxN	2 hr,<br>n= 8;<br>6 hr,<br>n=3	Seagrass +	Wales, UK	Fish / Juv+	*Zostera marina*	13	na	(Unsworth et al. 2014)
S-BRUV/ MaxN	30 min<br>n=54	Seagrass	Spain, Mediterranean Sea	Fish / Juv	*Posidonia oceanica*	Focused on *Diplodus annularis*	na	(Díaz-Gil et al. 2017)
S-BRUV/ MaxN	2 hr<br>n=28	Seagrass	North Wales, UK	Fish / Juv+	*Zostera marina*	20	na	(Peters et al. 2015)

Seine net	n=46	Seagrass +	North Wales, UK	Fish+ / Juv+	*Zostera marina*	19	na	(McCloskey and Unsworth 2015)
Seine net	n=28+16	Seagrass	North Wales, UK	Fish / Juv	*Zostera marina*	26	na	(Bertelli and Unsworth 2014)
Net & poison	n=54	Seagrass	NSW, Australia	Fish+ / Juv	*Zostera capricorni*	78	na	(Bell et al. 1988)
Seine net	n=48	Seagrass +	NSW, Australia	Fish / Juv	*Zostera capricorni*	35	na	(West and King 1996)
Seine net	n=48	Seagrass+	NSW, Australia	Fish / Juv	*Zostera capricorni*	49	na	(Gray et al. 1996)
Enclosure net	n=240	Seagrass +	Indian Ocean, Sri Lanka	Fish / Juv	*Halophila ovalis, Halodule pinifolia*	46	na	(Pinto and Punchihewa 1996)
Trawl	n=168	Seagrass +	Southern Gulf of Mexico	Fish / Juv	*Thalassia testudinum*	77	na	(Yáñez-Arancibia et al. 1993)
Seine net	n=180	Seagrass	NWS, Australia	Fish / Juv	*Zostera capricorni*	80	na	(Hannan and Williams 1998)
Seine and gill net	?	Seagrass +	Australia, Moreton Bay	Fish / Juv	*Zostera capricorni*	30	na	(Morton 1990)
Trawl	n=410	Seagrass	Caribbean, Panama	Fish / Juv	*Thalassia testudinum, Syringodium filiforme*	106	na	(Weinstein and Heck 1979)
Trawl	n=75(?)	Seagrass+	Massachusetts, USA	Fish+ / Juv	-	22	na	(Heck et al. 1989)
Trawl	n=336, 126	Seagrass	Florida, USA	Fish / Juv	*Thalassia testudinum, Syringodium filiforme, Halodule wrightii*	53 and 37	na	(Stoner 1983)
Thrown Trap & Gillnet	n=100, 18	Seagrass	Florida, USA	Fish / Juv	*Thalassia* sp.	71	na	(Sogard et al. 1989)
Drop sampler	n=13	Seagrass+	Guatemala, Caribbean	Fish+ / Juv	*Thalassia testudinum*	16	na	(Arrivillaga and Baltz 1999)
Drop, seine and trawl nets	n=43, 270, 498	Seagrass+	Bahamas, Caribbean	Fish / Juv	-	71	na	(Newman et al. 2007)
Seine and pop nets	n=42	Seagrass+	South Australia	Fish+ / Juv	*Zostera muelleri*	18	na	(Bloomfield and Gillanders 2005)
UVC	Tr 25 m n=388 & n=201	Seagrass+	Tanzania and Aruba	Fish / Juv	-	Focused on 2	na	(Dorenbosch et al. 2006)
Trawl & ring nets with poison	n= 18 and 4	Seagrass	Chesapeake Bay	Fish / Juv	*Ruppia maritima, Zostera marina*	36	na	(Weinstein and Brooks 1983)
Seine net	n=48	Seagrass+	Southern Australia	Fish / Juv	*Heterozostera tasmanica*	28	na	(Jenkins and Wheatley 1998)

UVC and net	Tr 300 m<br>n=42 (?)	Seagrass+	Bonaire	Fish / Juv	*Thalassia testudinum*	Focused on 16	Abundance: Net>VS	(Nagelkerken, van der Velde, et al. 2000)
UVC	Tr 50 m<br>n=131	Seagrass+	Caribbean, Curacao	Fish / Juv	*Thalassia testudinum*	47	na	(Nagelkerken, Dorenbosch, et al. 2000)
UVC	Tr 50 m n=12	Seagrass+	Caribbean, Belize	Fish / Juv	-	Focused on 6	na	(Mumby et al. 2004)
UVC	Tr 50 m<br>n=8(?)	Seagrass+	St. Croix, Virgin Islands	Fish / Juv	*Thalassia testudinum, Syringodium filiforme*	25	na	(Adams and Ebersole 2002)
Seine and trap net	n =32 & 96hrs	Seagrass	Caribbean, Guadalupe Is.	Fish / Juv	*Thalassia testudinum, Syringodium filiforme*	87	na	(Kopp et al. 2010)
UVC	Tr 50 m<br>n= 48 (?)	Seagrass+	Caribbean, Curacao	Fish / Juv	*Thalassia testudinum*	114 (all habitats)	na	(Nagelkerken and van der Velde 2002)
UVC	Tr 50 m<br>n=522	Seagrass+	Caribbean, Curacao	Fish / Juv	*Thalassia testudinum*	Focused on 9	na	(Cocheret de la Morinière et al. 2002)
UVC	Tr 50 m<br>n=120	Seagrass+	Caribbean<br>Puerto Rico	Fish / Juv	*Thalassia testudinum*	Focused on 20	na	(Aguilar-Perera and Appeldoorn 2007)
Net	n=20	Seagrass+	Pacific<br>Indonesia	Fish / Juv	*Thalassia hemprichii* (Ehrenberg), *Enhalus acoroides*	114	na	(Unsworth et al. 2008)
UVC	Tr 20 m<br>n= 3-7	Seagrass+	Philippines & PG	Fish / Juv	*Thalassia hemprichii, Halodule pinifolia, Cymodocea rotundata, T. hemprichii, Enhalus acoroides*	38	na	(Honda et al. 2013)
UVC	Qd 5x5 n=120	Seagrass+	Indian Ocean (Tanzania)	Fish / Juv+	Thalassodendron ciliatum, Enhalus acoroides, Thalassia hemprichii, Cymodocea rotundata.	36	na	(Dorenbosch et al. 2005)
UVC	Tr 20 m n= 32	Seagrass+	Mediterranean Sea	Fish / Juv+	*Posidonia oceanica*	24	na	(Guidetti 2000)
Net	n= 64	Seagrass	Mediterranean Sea	Fish+ / Juv	*Zostera marina*	31	na	(Jackson et al. 2006)
UVC	Tr 20 m<br>n= 7 x 6 x 4	Seagrass+	Japan	Fish / Juv+	14 species	62	na	(Nakamura and Tsuchiya 2008)
Seine net	N=46+16	Seagrass+	North Wales	Fish+ / Juv+	*Zostera marina*	19+17	na	(McCloskey and Unsworth 2015)
UVC	Tr 50 m<br>n=36	Cancun - Puerto Morelos	Mexico	Fish / Juv+	-	62	na	(Arias-González et al. 2008)
UVC	Tr 20 m<br>n= 48	Mahahual - Xcalak	Mexico	Fish / Juv+	-	28	na	(Yeager and Arias-Gonzalez 2008)

Bibliography

Adams, A.J. and Ebersole, J.P. 2002. Use of back-reef and lagoon habitats by coral reef fishes. *Marine Ecology Progress Series* 228, pp. 213–226.

Aguilar-Perera, A. and Appeldoorn, R.S. 2007. Variation in juvenile fish density along the mangrove–seagrass–coral reef continuum in SW Puerto Rico. *Marine Ecology Progress Series* 348, pp. 139–148.

Arias-González, J.E., Legendre, P. and Rodríguez-Zaragoza, F.A. 2008. Scaling up beta diversity on Caribbean coral reefs. *Journal of Experimental Marine Biology and Ecology* 366(1–2), pp. 28–36.

Arrivillaga, A. and Baltz, D.M. 1999. Comparison of Fishes and Macroinvertebrates on seagrass and bare-sand sites on Guatemala's Atlantic Coast. *Bulletin of Marine Science* 65(2), pp. 301–319.

Bell, J.D., Steffe, A.S. and Westoby, M. 1988. Location of seagrass beds in estuaries: effects on associated fish and decapods. *Journal of Experimental Marine Biology and Ecology* 122(2), pp. 127–146.

Bertelli, C.M. and Unsworth, R.K.F. 2014. Protecting the hand that feeds us: seagrass (Zostera marina) serves as commercial juvenile fish habitat. *Marine Pollution Bulletin* 83(2), pp. 425–429.

Bloomfield, A.L. and Gillanders, B.M. 2005. Fish and invertebrate assemblages in seagrass, mangrove, saltmarsh, and nonvegetated habitats. *Estuaries* 28(1), pp. 63–77.

Cocheret de la Morinière, E., Pollux, B.J.A., Nagelkerken, I. and van der Velde, G. 2002. Post-settlement Life Cycle Migration Patterns and Habitat Preference of Coral Reef Fish that use Seagrass and Mangrove Habitats as Nurseries. *Estuarine, Coastal and Shelf Science* 55(2), pp. 309–321.

Dalley, K.L., Gregory, R.S., Morris, C.J. and Cote, D. 2017. Seabed habitat determines fish and macroinvertebrate community associations in a subarctic marine coastal nursery. *Transactions of the American Fisheries Society* 146(6), pp. 1115–1125.

Díaz-Gil, C., Smee, S.L., Cotgrove, L., Follana-Berná, G., Hinz, H., Marti-Puig, P., Grau, A., Palmer, M. and Catalán, I.A. 2017. Using stereoscopic video cameras to evaluate seagrass meadows nursery function in the Mediterranean. *Marine biology* 164(6), p. 137.

Dorenbosch, M., Grol, M.G.G., Christianen, M.J.A., Nagelkerken, I. and van der Velde, G. 2005. Indo-Pacific seagrass beds and mangroves contribute to fish density and diversity on adjacent coral reefs. *Marine Ecology Progress Series* 302, pp. 63–76.

Dorenbosch, M., Grol, M.G.G., Nagelkerken, I. and van der Velde, G. 2006. Seagrass beds and mangroves as potential nurseries for the threatened Indo-Pacific humphead wrasse, Cheilinus undulatus and Caribbean rainbow parrotfish, Scarus guacamaia. *Biological Conservation* 129(2), pp. 277–282.

Elliott, S.A.M., Turrell, W.R., Heath, M.R. and Bailey, D.M. 2017. Juvenile gadoid habitat and ontogenetic shift observations using stereo-video baited cameras. *Marine Ecology Progress Series* 568, pp. 123–135.

Galaiduk, R., Radford, B.T., Wilson, S.K. and Harvey, E.S. 2017. Comparing two remote video survey methods for spatial predictions of the distribution and environmental niche suitability of demersal fishes. *Scientific reports* 7(1), p. 17633.

Gladstone, W., Lindfield, S., Coleman, M. and Kelaher, B. 2012. Optimisation of baited remote underwater video sampling designs for estuarine fish assemblages. *Journal of Experimental Marine Biology and Ecology* 429, pp. 28–35.

Gray, C.A., McElligott, D.J. and Chick, R.C. 1996. Intra- and inter-estuary differences in assemblages of fishes associated with shallow seagrass and bare sand. *Marine and Freshwater Research* 47(5), p. 723.

Guidetti, P. 2000. Differences Among Fish Assemblages Associated with Nearshore Posidonia oceanica Seagrass Beds, Rocky–algal Reefs and Unvegetated Sand Habitats in the Adriatic Sea. *Estuarine, Coastal and Shelf Science* 50(4), pp. 515–529.

Hannan, J.C. and Williams, R.J. 1998. Recruitment of juvenile marine fishes to seagrass habitat in a temperate australian estuary. *Estuaries* 21(1), p. 29.

Harvey, E.S., Cappo, M., Kendrick, G.A. and McLean, D.L. 2013. Coastal fish assemblages reflect geological and oceanographic gradients within an Australian zootone. *Plos One* 8(11), p. e80955.

Heck, K.L., Able, K.W., Fahay, M.P. and Roman, C.T. 1989. Fishes and Decapod Crustaceans of Cape Cod Eelgrass Meadows: Species Composition, Seasonal Abundance Patterns and Comparison with Unvegetated Substrates. *Estuaries* 12(2), p. 59.

Honda, K., Nakamura, Y., Nakaoka, M., Uy, W.H. and Fortes, M.D. 2013. Habitat use by fishes in coral reefs, seagrass beds and mangrove habitats in the Philippines. *Plos One* 8(8), p. e65735.

Jackson, E.L., Attrill, M.J. and Jones, M.B. 2006. Habitat characteristics and spatial arrangement affecting the diversity of fish and decapod assemblages of seagrass (Zostera marina) beds around the coast of Jersey (English Channel). *Estuarine, Coastal and Shelf Science* 68(3–4), pp. 421–432.

Jenkins, G.P. and Wheatley, M.J. 1998. The influence of habitat structure on nearshore fish assemblages in a southern Australian embayment: Comparison of shallow seagrass, reef-algal and unvegetated sand habitats, with emphasis on their importance to recruitment. *Journal of Experimental Marine Biology and Ecology* 221(2), pp. 147–172.

Jones B., Cullen-Unsworth L.C., Howard R., Unsworth R.K.F. 2018. Complex yet fauna-deficient seagrass ecosystems at risk in southern Myanmar. *Botanica Marina* 61(3), pp. 193-203.

Kopp, D., Bouchon-Navaro, Y., Louis, M., Mouillot, D. and Bouchon, C. 2010. Juvenile fish assemblages in caribbean seagrass beds: does nearby habitat matter? *Journal of Coastal Research* 26, pp. 1133–1141.

McCloskey, R.M. and Unsworth, R.K.F. 2015. Decreasing seagrass density negatively influences associated fauna. *PeerJ* 3, p. e1053.

Morton, R.M. 1990. Community structure, density and standing crop of fishes in a subtropical Australian mangrove area. *Marine biology* 105(3), pp. 385–394.

Mumby, P.J., Edwards, A.J., Arias-González, J.E., Lindeman, K.C., Blackwell, P.G., Gall, A., Gorczynska, M.I., Harborne, A.R., Pescod, C.L., Renken, H., Wabnitz, C.C.C. and Llewellyn, G. 2004. Mangroves enhance the biomass of coral reef fish communities in the Caribbean. *Nature* 427(6974), pp. 533–536.

Nagelkerken, I., Dorenbosch, M., Verberk, W., Cocheret de la Morinière, E. and van der Velde, G. 2000. Importance of shallow-water biotopes of a Caribbean bay for juvenile coral reef fishes: patterns in biotope association, community structure and spatial distribution. *Marine Ecology Progress Series* 202, pp. 175–192.

Nagelkerken, I. and van der Velde, G. 2002. Do non-estuarine mangroves harbour higher densities of juvenile fish than adjacent shallow-water and coral reef habitats in Curaçao (Netherlands Antilles)? *Marine Ecology Progress Series* 245, pp. 191–204.

Nagelkerken, I., van der Velde, G., Gorissen, M.W., Meijer, G.J., Van't Hof, T. and den Hartog, C. 2000. Importance of mangroves, seagrass beds and the shallow coral reef as a nursery for important coral reef fishes, using a visual census technique. *Estuarine, Coastal and Shelf Science* 51(1), pp. 31–44.

Nakamura, Y. and Tsuchiya, M. 2008. Spatial and temporal patterns of seagrass habitat use by fishes at the Ryukyu Islands, Japan. *Estuarine, Coastal and Shelf Science* 76(2), pp. 345–356.

Newman, S.P., Handy, R.D. and Gruber, S.H. 2007. Spatial and temporal variations in mangrove and seagrass faunal communities at Bimini, Bahamas. *Bulletin of Marine Science* 80(3), p. 529–553,.

Pelletier, D., Leleu, K., Mallet, D., Mou-Tham, G., Hervé, G., Boureau, M. and Guilpart, N. 2012. Remote high-definition rotating video enables fast spatial survey of marine underwater macrofauna and habitats. *Plos One* 7(2), p. e30536.

Perry, D., Staveley, T.A.B. and Gullström, M. 2018. Habitat Connectivity of Fish in Temperate Shallow-Water Seascapes. *Frontiers in Marine Science* 4.

Peters, J.R., McCloskey, R.M., Hinder, S.L. and Unsworth, R.K.F. 2015. Motile fauna of sub-tidal Zostera marina meadows in England and Wales. *Marine Biodiversity* 45(4), pp. 647–654.

Pinto, L. and Punchihewa, N.N. 1996. Utilisation of mangroves and seagrasses by fishes in the Negombo Estuary, Sri Lanka. *Marine biology* 126(2), pp. 333–345.

Sogard, S.M., Powell, G.V.N. and Holmquist, J.G. 1989. Spatial Distribution and Trends in Abundance Of Fishes Residing in Seagrass Meadows on Florida Bay Mudbanks. *Bulletin of Marine Science* 44(1), pp. 179–199.

Stat M.,Jeffrey J.,DiBattista JD.,Newman S.J., Bunce M, Harvey E. 2018. Combined use of eDNA metabarcoding and video surveillance for the assessment of fish biodiversity. *Conservation Biology*, 33 (1), pp. 196-205.

Stoner, A.W. 1983. Distribution of fishes in seagrass meadows: role of macrophyte biomass and species composition. *Fishery Bulletin- National Oceanic and Atmospheric Administration*.

Unsworth, R.K.F., De León, P.S., Garrard, S.L., Jompa, J., Smith, D.J. and Bell, J.J. 2008. High connectivity of Indo-Pacific seagrass fish assemblages with mangrove and coral reef habitats. *Marine Ecology Progress Series* 353, pp. 213–224.

Unsworth, R.K.F., Peters, J.R., McCloskey, R.M. and Hinder, S.L. 2014. Optimising stereo baited underwater video for sampling fish and invertebrates in temperate coastal habitats. *Estuarine, Coastal and Shelf Science* 150, pp. 281–287.

Weinstein, M.P. and Brooks, H.A. 1983. Comparative ecology of nekton residing in a tidal creek and adjacent seagrass meadow: community composition and structure. *Marine Ecology Progress Series*.

Weinstein, M.P. and Heck, K.L. 1979. Ichthyofauna of seagrass meadows along the Caribbean coast of Panama and in the Gulf of Mexico: Composition, structure and community ecology. *Marine biology* 50(2), pp. 97–107.

West, R.J. and King, R.J. 1996. Marine, brackish, and freshwater fish communities in the vegetated and bare shallows of an australian coastal river. *Estuaries* 19(1), p. 31.

Whitmarsh, S.K., Fairweather, P.G., Brock, D.J. and Miller, D. 2014. Nektonic assemblages determined from baited underwater video in protected versus unprotected shallow seagrass meadows on Kangaroo Island, South Australia. *Marine Ecology Progress Series* 503, pp. 205–218.

Yáñez-Arancibia, A., Lara-Domínguez, A.L. and Day, J.W. 1993. Interactions between mangrove and seagrass habitats mediated by estuarine nekton assemblages: coupling of primary and secondary production. *Hydrobiologia* 264(1), pp. 1–12.

Yeager, L.A. and Arias-Gonzalez, J.E. 2008. Preliminary Survey of Fish Community Composition in Seagrass Habitat in Two Back-Reef Lagoons of the Southern Mexican Caribbea

Supplementary Table S2. Abundances (mean ± se) detected during the present study by Remote Underwater Video (RUV) and Underwater Visual Census (UVC) of juvenile and adult fish species inhabiting two seagrass habitats located in the reef lagoon of Puerto Morelos (Cancun, Mexico): Backreef and Lagoon.

Species	Fisheries importance	Aquarium importance	Trophic Group Diet	Habitat Preference	UVC				RUV			
					Backreef		Lagoon		Backreef		Lagoon	
					Juvenile	Adult	Juvenile	Adult	Juvenile	Adult	Juvenile	Adult
Halichoeres maculipinna		Commercial	Benthic Carnivore: invertebrates	Reef	0.15±0.10							
Halichoeres pictus		Commercial	Benthic Carnivore: invertebrates	Reef					0.08±0.08			
Chaetodon striatus		Commercial	Benthic Carnivore: invertebrates	Reef	0.38±0.38							
Chaetodon capistratus		Commercial	Benthic Carnivore: invertebrates	Reef			0.07±0.07					
Acanthostracion polygonius	Commercial	Commercial	Benthic Carnivore: invertebrates	Reef						0.15±0.10		0.07±0.07
Ulaema lefroyi	Minor commercial & bait		Benthic Carnivore: invertebrates	Seagrass						2.69±1.65		
Xyrichtys splendens		Commercial	Benthic Carnivore: invertebrates	Seagrass		0.31±0.31			0.08±0.08	0.38±0.24	0.29±0.16	
Xyrichtys novacula	Minor commercial & gamefish	Commercial	Benthic Carnivore: invertebrates	Seagrass						0.23±0.23		0.29±0.16
Gerres cinereus	Minor commercial & bait		Benthic Carnivore: invertebrates	Seagrass & Reef								0.21±0.15
Calamus bajonado	Minor commercial & gamefish		Benthic Carnivore: invertebrates	Seagrass & Reef							0.07±0.07	0.07±0.07
Sphoeroides testudineus	No interest		Benthic Carnivore: invertebrates	Seagrass & Reef				0.07±0.07				
Monacanthus tuckeri	No interest		Benthic Carnivore: invertebrates	Seagrass & Reef							0.43±0.17	0.21±0.15
Diodon holocanthus	Minor commercial	Commercial	Benthic Carnivore: invertebrates	Seagrass & Reef			0.07±0.07					0.07±0.07
Balistes vetula	Minor commercial & gamefish	Commercial	Benthic Carnivore: invertebrates	Seagrass & Reef						0.08±0.08		
Acanthostracion quadricornis	Minor commercial	Commercial	Benthic Carnivore: invertebrates & macrophytes	Seagrass & Reef					0.08±0.08			0.14±0.14
Hypoplectrus .sp		Commercial	Benthic Carnivore: invertebrates & nekton	Reef							0.71±0.71	
Aulostomus maculatus	Minor commercial	Commercial	Benthic Carnivore: invertebrates & nekton	Reef						0.08±0.08		
Halichoeres poeyi		Commercial	Benthic Carnivore: invertebrates & nekton	Seagrass	0.62±0.33				0.92±0.38		0.36±0.13	
Hypanus americanus	Commercial & gamefish		Benthic Carnivore: invertebrates & nekton	Seagrass & Reef						0.38±0.14		0.07±0.07
Halichoeres bivittatus		Commercial	Benthic Carnivore: invertebrates & nekton	Seagrass & Reef	11.62±1.94	0.15±0.10	2.57±0.86	1.0±0.85	5.69±1.36	1.54±0.53	4.64±1.0	2.0±0.70
Thalassoma bifasciatum		Commercial	Benthic Carnivore: invertebrates & nekton	Seagrass & Reef	0.23±0.12		0.07±0.07					

Pseudopeneus maculatus	Commercial	Commercial	Benthic Carnivore: invertebrates & nekton	Seagrass & Reef				0.07±0.07		0.85±0.77	1.79±1.42	1.07±0.85
Urobatis jamaicensis		Public aquariums	Benthic Carnivore: invertebrates & nekton	Seagrass & Reef			0.07±0.07	0.07±0.07		0.08±0.08		0.29±0.16
Stephanolepis hispidus	Subsistence fisheries	Commercial	Benthic Omnivore: Invertebrates & seaweed	Seagrass						0.08±0.08	0.14±0.10	0.21±0.21
Stegastes leucostictus		Commercial	Benthic Omnivore: Invertebrates & seaweed	Seagrass & Reef	0.23±0.17							
Monacanthus ciliatus	Subsistence fisheries	Commercial	Benthic Omnivore: Invertebrates & seaweed	Seagrass & Reef			0.07±0.07			0.08±0.08	0.43±0.36	0.36±0.13
Lactophrys triqueter	Minor commercial	Commercial	Benthic Omnivore: invertebrates & macrophytes	Reef						0.08±0.08		
Canthigaster jamestyleri	No interest		Benthic Omnivore: invertebrates & macrophytes	Seagrass & Reef	0.08±0.08						0.07±0.07	0.07±0.07
Sphoeroides spengleri		Commercial	Benthic Omnivore: invertebrates & macrophytes	Seagrass & Reef						0.23±0.12	0.07±0.07	0.14±0.10
Canthingaster rostata		Commercial	Benthic Omnivore: invertebrates & macrophytes	Seagrass & Reef	0.08±0.08				0.23±0.17	0.08±0.08	0.07±0.07	
Lactophrys bicaudalis		Commercial	Benthic Omnivore: invertebrates & macrophytes	Seagrass & Reef						0.38±0.18		0.36±0.17
Lactophrys trigonus	Commercial	Commercial	Benthic Omnivore: invertebrates & macrophytes	Seagrass & Reef		0.08±0.08				1.08±0.29		0.79±0.15
Pareques acuminatus	Minor commercial	Commercial	Benthic Omnivore: invertebrates & macrophytes	Seagrass & Reef	0.38±0.21		0.14±0.10					
Scomberomorus regalis	Minor commercial & gamefish		Benthopelagic Carnivore: invertebrates & nekton	Reef						0.08±0.08		
Ocyurus chrysurus	Commercial, aquaculture & gamefish	Public aquariums	Benthopelagic Carnivore: invertebrates & nekton	Reef			7.07±6.99			0.38±0.38	5.71±3.75	1.86±1.39
*Sepioteuthis sepioidea**			Benthopelagic Carnivore: invertebrates & nekton	Seagrass & Reef								
Remora remora	Commercial		Benthopelagic Carnivore: invertebrates & nekton	Seagrass & Reef								0.14±0.14
Clupeidae .sp	Commercial & bait		Benthopelagic Carnivore: invertebrates & nekton	Seagrass & Reef								0.21±0.21

Caranx ruber	Commercial & gamefish		Benthopelagic Carnivore: invertebrates & nekton	Seagrass & Reef				1±1		2.46±0.92		2.93±1.31
Carangoides bartholomaei	Commercial & gamefish		Benthopelagic Carnivore: invertebrates & nekton	Seagrass & Reef				0.57±0.57		0.15±0.10		0.50±0.34
Aetobatus narinari	Minor commercial & gamefish		Benthopelagic Carnivore: invertebrates & nekton	Seagrass & Reef			0.07±0.07					
Caranx crysos	Minor commercial & gamefish		Benthopelagic Carnivore: invertebrates & nekton	Seagrass & Reef								0.07±0.07
Sphyraena borealis	Minor commercial & gamefish		Benthopelagic Carnivore: invertebrates & nekton	Seagrass & Reef								2.71±2.71
Canthidermis sufflamen	Commercial & gamefish	Commercial	Benthopelagic Carnivore: invertebrates	Seagrass & Reef						0.54±0.18		0.14±0.10
Lutjanus analis	Highly commercial & gamefish	Commercial	Benthopelagic Carnivore: invertebrates & nekton	Seagrass & Reef						0.08±0.08		
Haemulon flavolineatum	Commercial & bait	Public aquariums	Benthopelagic Carnivore: invertebrates	Seagrass & Reef			0.29±0.29					
Trachinotus falcatus	Commercial, aquaculture & gamefish	Public aquariums	Benthopelagic Carnivore: invertebrates & nekton	Seagrass & Reef						0.54±0.24		
Haemulon melanurum	Minor commercial	Public aquariums	Benthopelagic Carnivore: invertebrates	Seagrass & Reef			0.07±0.07					
Haemulon parra	Minor commercial	Public aquariums	Benthopelagic Carnivore: invertebrates & nekton	Seagrass & Reef	0.54±0.54							
Haemulon aurolineatum	Minor commercial & bait	Public aquariums	Benthopelagic Carnivore: invertebrates	Seagrass & Reef							10.29±10.29	
Haemulon plumieri	Minor commercial & gamefish	Public aquariums	Benthopelagic Carnivore: invertebrates & nekton	Seagrass & Reef			1.79±1.06		0.23±0.23	0.08±0.08	0.57±0.34	0.07±0.07
Sphyraena barracuda	Minor commercial & gamefish	Public aquariums	Benthopelagic Carnivore: invertebrates & nekton	Seagrass & Reef						0.08±0.08		0.07±0.07
Pomacanthus paru	Minor commercial	Commercial	Benthopelagic Omnivore: Invertebrates & seaweed	Reef	0.08±0.08		0.07±0.07					
*Chelonia mydas**			Benthopelagic Omnivore:	Seagrass & Reef								

			invertebrates & macrophytes									
Aluterus monoceros	Commercial		Benthopelagic Omnivore: Invertebrates & seaweed	Seagrass & Reef								0.07±0.07
Acanthurus tractus	Minor commercial & bait	Commercial	Herbivore: macrophytes	Reef	1.69±0.61				0.23±0.17			
Cryptotomus roseus		Commercial	Herbivore: macrophytes	Seagrass					0.23±0.17	0.15±0.10	0.36±0.29	0.07±0.07
Sparisoma viride	Minor commercial	Commercial	Herbivore: macrophytes	Seagrass					0.08±0.08		0.21±0.15	
Sparisoma radians	Potential interest	Commercial	Herbivore: macrophytes	Seagrass	0.31±0.24				0.62±0.29	0.38±0.31	0.50±0.37	0.07±0.07
Sparisoma atomarium		Commercial	Herbivore: macrophytes	Seagrass & Reef			0.14±0.14				0.43±0.23	
Sparisoma rubripinne	Minor commercial	Commercial	Herbivore: macrophytes	Seagrass & Reef								0.07±0.07
Sparisoma chrysopterum	Minor commercial	Commercial	Herbivore: macrophytes	Seagrass & Reef		0.08±0.08				0.46±0.27		
Scarus iseri	Minor commercial	Commercial	Herbivore: macrophytes	Seagrass & Reef	0.08±0.08		0.43±0.36				0.07±0.07	

Supplementary Table S3. PERMANOVA results for species and family richness between methods (UVC & RUV) and habitats (Backreef and Lagoon) Location as random factor.

PERMANOVA table of results for species richness

```
                                                Unique
Source  df     SS     MS  Pseudo-F  P(perm)    perms
Me       1  27724  27724    38.467   0.0001     9953
Ha       1 908.18 908.18    1.2601    0.297     9944
Lo       2 3205.1 1602.6    2.2236   0.0409     9928
MexHa    1  842.7  842.7    1.1693   0.3356     9943
Res     48  34594 720.72
Total 53    67404
```

PERMANOVA table of results for families' richness

```
                                                Unique
Source  df     SS     MS  Pseudo-F  P(perm)    perms
Me       1  21481  21481    37.453   0.0001     9954
Ha       1 918.67 918.67    1.6017      0.2     9945
Lo       2 1811.3 905.65     1.579   0.1584     9951
MexHa    1 624.22 624.22    1.0883   0.3554     9952
Res     48  27530 573.55
Total 53    52424
```

Supplementary Table S4. PERMANOVA results for juvenile and adult fish abundances between methods (UVC & RUV) and habitats (Backreef and Lagoon) with Location as random factor.

PERMANOVA table of results for juvenile fish abundances

Source	df	SS	MS	Pseudo-F	P(perm)	Unique perms
Me	1	2050.5	2050.5	2.8768	0.0467	9954
Ha	1	2073.5	2073.5	2.9091	0.0374	9938
Lo	2	6162.2	3081.1	4.3227	0.0021	9955
**MexHa**	**1**	**2728.2**	**2728.2**	**3.8275**	**0.0178**	**9946**
Res	48	34213	712.77			
Total	53	47382				

PERMANOVA table of results for adult fish abundances

Source	df	SS	MS	Pseudo-F	P(perm)	Unique perms
**Me**	**1**	**55028**	**55028**	**50.653**	**0.0001**	**9942**
Ha	1	1481.5	1481.5	1.3637	0.2371	9945
Lo	2	3303.7	1651.8	1.5205	0.2086	9955
MexHa	1	1570.9	1570.9	1.446	0.228	9937
Res	48	52146	1086.4			
Total	53	1.1312E5				

Supplementary Table S5. PERMANOVA results for benthic carnivores fish abundances between methods (UVC & RUV) and habitats (Backreef and Lagoon) with Location as random factor.

PERMANOVA table of results for abundance of benthic carnivores

Source	df	SS	MS	Pseudo-F	P(perm)	Unique perms
Me	1	3484.1	3484.1	5.426	0.0015	9958
Ha	1	3500.6	3500.6	5.4516	0.003	9959
Lo	2	2690.4	1345.2	2.0949	0.0748	9961
**MexHa**	**1**	**3447.3**	**3447.3**	**5.3687**	**0.0012**	**9956**
Res	48	30821	642.11			
Total	53	44061				

PERMANOVA table of results for abundance of benthic herbivores

Source	df	SS	MS	Pseudo-F	P(perm)	Unique perms
**Me**	**1**	**16210**	**16210**	**13.179**	**0.0001**	**9951**
**Ha**	**1**	**6174.9**	**6174.9**	**5.0203**	**0.016**	**9942**
Lo	2	7126.6	3563.3	2.897	0.0321	9949
MexHa	1	1825	1825	1.4838	0.2189	9949
Res	48	59039	1230			
Total	53	90361				

PERMANOVA table of results for benthopelagic carnivores

Source	df	SS	MS	Pseudo-F	P(perm)	Unique perms
**Me**	**1**	**37369**	**37369**	**24.549**	**0.0001**	**9944**
Ha	1	1225	1225	0.8048	0.4381	9936
Lo	2	6000.1	3000	1.9709	0.1106	9953
MexHa	1	3810.7	3810.7	2.5034	0.0899	9940
Res	48	73064	1522.2			
Total	53	1.2081E5				
